# Special Issue “Diabetic Nephropathy: Diagnosis, Prevention and Treatment”

**DOI:** 10.3390/jcm9030813

**Published:** 2020-03-17

**Authors:** Marta Ruiz-Ortega, Raul R. Rodrigues-Diez, Carolina Lavoz, Sandra Rayego-Mateos

**Affiliations:** 1 Molecular and Cellular Biology in Renal and Vascular Pathology, IIS-Fundación Jiménez Díaz-UniversidadAutónoma Madrid, 28040 Madrid, Spain; rrodrigues@fjd.es; 2 Red de Investigación Renal (REDINREN), Instituto de Salud Carlos III, 28029 Madrid, Spain; 3 Division of Nephrology, School of Medicine, Universidad Austral de Chile, 5090000 Valdivia, Chile; carolina.lavoz@uach.cl; 4 Vascular and Renal Translational Research Group. Institut de Recerca Biomèdica de Lleida (IRBLleida), 25198 Lleida, Spain; srayego@fjd.es

**Keywords:** chronic kidney disease, diabetic nephropathy, biomarkers, treatment

## Abstract

Diabetic nephropathy (DN) is the main cause of end-stage renal disease. DN is a complex disease mediated by genetic and environmental factors, and many cellular and molecular mechanisms are involved in renal damage in diabetes. There are no biomarkers that reflect the severity of the underlying renal histopathological changes and can effectively predict the progression of renal damage and stratify the risk of DN among individuals with diabetes mellitus. Current therapeutic strategies are based on the strict control of glucose and blood pressure levels and, although there are new anti-diabetic drugs, these treatments only retard renal damage progression, being necessary novel therapies. In this Special Issue, there are several comprehensive reviews and interesting original papers covering all these topics, which would be of interest to the growing number of readers of the *Journal of Clinical Medicine*.

Chronic kidney disease (CKD) is a major health problem because of its association with a high cardiovascular risk, and most CKD patients progress to end-stage renal disease (ESRD), requiring dialysis or transplantation. Type 2 diabetes mellitus is one of the most important risk factors in the development of CKD, around 30%–50% of ESRD patients worldwide come from a diabetic origin. Diabetic nephropathy (DN) has been classically considered as a metabolic disease; however, increasing data support the important role of immune response in the pathogenesis of this disease. Here, we provide the reader with four comprehensive reviews about the molecular mechanisms involved in renal damage in diabetes, pointing out novel therapeutic options for this disease that would be of interest to general medical readers as well as specialists in diabetes. The article of Donate-Correa et al. [1] delves into the pathogenesis of proinflammatory molecules and mechanisms related to the development and progression of DN. The authors discuss the potential utility of agents that target inflammatory-related factors or pathways, including inflammatory cytokines, oxidative stress or pro-inflammatory pathways, such as Signal transducers and activator of transcription (STAT/JAK) or Nuclear Factor-кB, to be used as new therapeutic targets in this pathology. However, they remarked on the necessity of perform new clinical trials to examine the potential renoprotective efficacy of these approaches in the context of DN. In this sense, Lavoz et al. [2] highlighted the importance of one of these inflammatory processes, the Th17 immune response, in the pathogenesis of diabetic renal injury. In this article, authors reviewed the current information about the involvement of Th17/IL-17A in diabetes and diabetes-induced end-organ, with special attention to the kidney. They postulated reasonably the possible use of antibodies against IL-17A as an additional therapy in patients with DN. Other well-defined mechanisms implicated in the progression of diabetic complications are oxidative stress and extracellular matrix (ECM) accumulation. Concerning oxidative stress, Caro-Ordieres et al. [3] explain the antioxidant effects of natural antioxidant compounds, flavonoids, and remark on their anti-inflammatory and anti-diabetic properties. They review the recent pre-clinical and clinical investigations about the use of flavonoids to ameliorate diabetic complications. On the other hand, Garcia-Fernandez et al. [4] focused their review in the importance of the balance between the levels of matrix metalloproteinases (MMPs) and their inhibitors (TIMPs) in maintaining renal ECM homeostasis, as well as their contribution to ECM accumulation in DN. Although there is already a wide range of articles studying MMPs/TIMPs, the authors highlight the existence of some contradictory results, which manifest the complex regulation of MMPs, TIMPs, proinflammatory and profibrotic factors in this pathology.

One important problem is the growing number of DN patients, which highlights the urgent need for novel biomarkers that allow an earlier diagnosis of renal damage, as well as the identification of patients that rapidly progress to ERSD. In this Special Issue, there are four original papers about diagnostic methods in DN. De Bruyne et al.’s [5] study describes a new method to gain insight into biochemical changes occurring in renal cortical tissue before histological damage can be detected by conventional pathology. The method consists of the innovative application of near-infrared spectroscopy to renal biopsies in an objective and nondestructive way that could be used on routine stained tissue sections. However, large-scale prospective follow-up studies will be indispensable to translate their findings into the clinic. They found some spectral changes related to carbamoylation and glycation reactions, showing a biochemical signature associated with DN, and suggest that this method could be a useful tool to complement histopathological analysis, especially in post-transplant surveillance kidney biopsies. Another controversial point in the management of DN is the definition of renal function. In clinical practice, the renal function is calculated using different mathematical algorithms, including several variables, such as serum creatinine or cystatin-C levels, weight, height, and gender, leading to an estimated glomerular filtration rate (GFR). The study developed by Luis-Lima et al. [6] showed the relevance of the correct GFR estimation in diabetic patients. They demonstrated that the estimated GFR shows around a 30% error compared to measured GFR. Moreover, this error is even higher in patients with measured GFR less than 60 mL/min. Moreover, 25% of patients with hyperfiltration have the wrong diagnosis and 30% of DN patients are not located in the correct CKD stage. Authors demonstrate that the actual creatinine and/or cystatin-C based formulas in type 2 diabetes failed to properly reflect the renal function in diabetic patients in a wide range of GFR (from advanced CKD to hyperfiltration). Actually, microalbuminuria is one of the most used non-invasive diagnosis urinary biomarkers in DN to identify renal function decline. However, better urinary biomarkers are needed. In the manuscript of Koziolek et al. [7], a urinary proteomics approach was used to identify small proteins and peptides associated with pathological changes at first stage of the diabetic disease in a big cohort (563 diabetic patients). They identified several differentially expressed proteins between DN and healthy controls, including apolipoprotein A-I, beta-2-microglobulin, epithelial cadherin (E-cadherin) and lithostathine-1-alpha. Among them, they identified E-cadherin as a key protein in diabetes-induced renal damage and showed that its urinary excretion levels had enough sensibility to discriminate between the CKD different stages, suggesting E-cadherin as a potential urinary biomarker for diabetic patients. On the other hand, cardiovascular events are the main cause of death in DN patients; therefore, the definition of biomarkers of cardiovascular risk is very important. Vascular calcification (VC) has been identified as one of the main predictors of cardiovascular risk in CKD. However, there are no vascular calcification biomarkers for later diagnostic and early therapeutic strategy implementation in CKD. Vascular calcification consists of calcium phosphate mineral deposition in the intima and media layers of the vessels, characterized by deregulation of endogenous calcification inhibitors, abnormal mineral metabolism and inflammation. Previous studies have described the circulating vitamin K-dependent protein (VKDP), named Gla-rich protein (GRP), as a molecule with anti-calcifying and anti-inflammatory properties in the cardiovascular system, but there are no previous studies about its role in VC diagnostics. The study of Silva et al. [8] identified GRP as an early marker of vascular damage in CKD. In a cohort of 80 diabetic patients, authors demonstrated a close relationship between a decrease of serum GRP levels and the progression of renal damage from mild CKD (stage two) to moderate CKD (stage four) measured by estimated GFR. In addition, there was a negative correlation with markers of mineral metabolism (phosphate, Ca, CaxP, parathyroid hormone, Fibroblast growth factors-23), vascular calcification score, pulse pressure and IL-6 levels. They suggest the potential utility of GRP as an early marker of vascular damage in CKD.

Recognized therapeutic strategies used in DN patients, including the strict control of glucose levels, blood pressure, and new anti-diabetic drugs, only retard renal damage progression, but there are no novel direct therapies for DN. In the original paper by Lee at al. [9] they evaluate the relationship between long-term use of Beta2-Adrenergic Receptor Agonists (β2AR) and diabetic vascular complications in a cohort of Korean patients. Authors indicate that macro- and micro-vascular complications in diabetic patients decreased with an increased duration of β2AR agonist administration, exercising a protective effect in this population. Increasing studies are evaluating novel therapies based in plants used in Chinese traditional medicine, with promising data. In this sense, Do et al. [10] discuss the therapeutic use of Lespedeza bicolor (a member of the leguminosae family) to prevent familiar diabetic nephropathy. In this study, they developed several in vitro studies and in vivo models of diabetic-induced renal damage, using a precursor in advanced glycation end-products (AGEs) formation, the methylglyoxal (MGO). Authors provide evidence of how Lespedeza bicolor recovers MGO-induced metabolic dysfunction and glucotoxicity, and its associated mechanisms that regulate the formation of AGEs and cellular oxidative stress response. Importantly, a barrier to progress in the knowledge of DN is the lack of animal models resembling the main features of human DN, making more studies necessary. In the paper of Lavoz et al. [11], authors used the model of leptin-deficient BTBR ob/ob mice, a mouse model characterized by a kidney disease that mimics key features of advanced human DN and showed evidence of the reversibility of glomerular lesions. Therefore, this model has been recommended as an excellent approach for carrying out preclinical studies of therapeutic interventions. Using this model, Lavoz et al. found that vascular endothelial growth factor receptor-2 (VEGFR2 kinase inhibition therapeutic treatment, starting after kidney disease developed, reversed the structural abnormalities of DN, including the amelioration of mesangial matrix accumulation and renal inflammation mitigation, and also improved renal function. This group and others have proposed the developmental gene GREMLIN as a DN therapeutic target. Lavoz et al. have previously described that GREMLIN acts via VEGFR2 in tubular epithelial cells. Now, they have found that GREMLIN expression levels, but not the canonical VEGF ligands, were upregulated coincidentally with the onset of renal damage, and remained elevated thereafter in BTBR ob/ob mice. They proposed that the VEGFR2 blockade could be a potential therapeutic option for DN, mainly by targeting GREMLIN.
